# Combined Transpetrosal Approach for Salvage Surgery in the Treatment of Recurrent and Advanced Nasopharyngeal Carcinoma: A Case Report

**DOI:** 10.7759/cureus.84111

**Published:** 2025-05-14

**Authors:** Stephanie Suyhogo, Ness Jerold Justo, John Albert Dy

**Affiliations:** 1 Neurological Surgery, The Medical City, Pasig, PHL

**Keywords:** cerebral metastasis, combined petrosectomy, nasopharyngeal carcinoma (npc), retrosigmoid, transpetrosal approach

## Abstract

A 45-year-old female patient was diagnosed with nasopharyngeal carcinoma (NPC). She previously underwent chemotherapy and radiotherapy but now presents with multiple craniopathies and persistent severe headaches that significantly impair her daily activities due to local tumor progression. Notably, she had not been offered prior surgical intervention.

Salvage surgical therapy following maximal chemotherapy and radiotherapy is a viable option for patients with local tumor progression and signs of brain compression, aiming to relieve increased intracranial pressure through tumor debulking. In this case report, we advocate for a multi-corridor approach - combining anterior and posterior petrosectomy with a retrosigmoid craniotomy - to optimize tumor resection.

The choice of surgical approach is tailored to the extent of tumor invasion within the skull base, with the primary goal of achieving a safe and maximal resection. By utilizing multiple surgical corridors, extradural and intradural tumor burden can be effectively reduced. Mastery of various skull base approaches is essential to develop a comprehensive surgical repertoire, allowing for strategic combinations that optimize resection. Additionally, continuous monitoring and postoperative multidisciplinary care are crucial in guiding the next steps for these highly morbid conditions. In this case, the patient was discharged with a significant reduction in headache and no new neurological deficits. Salvage surgery remains a viable palliative option that can enhance the patient’s quality of life.

## Introduction

Advanced locoregional disease with skull base extension is an uncommon manifestation of nasopharyngeal carcinoma (NPC). Patients presenting with skull base involvement typically exhibit persistent, locally advanced disease. According to the 2024 guidelines issued by the National Comprehensive Cancer Network (NCCN) [1], the standard management for advanced locoregional or persistent NPC consists of radiotherapy in combination with systemic therapy or, alternatively, best supportive care. Surgical resection is not routinely recommended, and reports of salvage surgical interventions with palliative intent remain limited in the literature. Furthermore, certain anatomical involvements - including extensive intradural invasion, cavernous sinus infiltration, and pharyngobasilar fascia compromise - are frequently cited as contraindications to salvage surgery.

Salvage surgical therapy following maximal chemotherapy and radiotherapy is a viable option for patients with local tumor progression and signs of brain compression, aiming to relieve increased intracranial pressure through tumor debulking. In this case report, we advocate for a multi-corridor approach, combining anterior and posterior petrosectomy with a retrosigmoid craniotomy, to optimize tumor resection.

The choice of surgical approach should be tailored to the extent of tumor invasion within the skull base, with the primary goal of achieving a safe and maximal resection. Cao et al. [2] identified several high-risk regions for tumor dissemination, including the sphenoid bone, prestyloid compartment, prevertebral muscles, foramen lacerum, medial pterygoid plate, sphenoidal sinus, clivus, petrous apex, and foramen ovale. By utilizing multiple surgical corridors, extradural and intradural tumor burden can be effectively reduced. We report a case of NPC with skull base and cavernous sinus extension in a patient for whom all nonsurgical therapeutic modalities had been exhausted. A single-stage, combined supratentorial and infratentorial approach was employed as a salvage procedure to alleviate compressive symptoms while preserving vital neurovascular structures, including the cochlear and optic nerves, thereby maintaining the patient’s quality of life. This comprehensive approach enabled access to critical anatomical corridors-namely, the middle cranial fossa, cerebellopontine angle, and cavernous sinus-facilitating effective tumor debulking. 

Mastery of various skull base approaches is essential to develop a comprehensive surgical repertoire, allowing for strategic combinations that optimize resection. Additionally, continuous monitoring and postoperative multidisciplinary care are crucial in guiding the next steps for these highly morbid conditions. Salvage surgery remains a viable palliative option that can enhance the patient’s quality of life.

## Case presentation

The patient is a 45-year-old Filipino female with a confirmed diagnosis of NPC, initially diagnosed in 2011 via tissue biopsy following the presentation of a left-sided nasopharyngeal mass. She underwent a treatment regimen comprising three cycles of cisplatin-based chemotherapy and 37 sessions of radiotherapy, resulting in initial remission.

In 2018, disease recurrence was identified in the right nasopharyngeal region through PET-CT imaging. Subsequent management included eight cycles of systemic chemotherapy consisting of gemcitabine, carboplatin, and denosumab, in addition to further radiotherapy and immunotherapy with cetuximab. No surgical intervention was pursued during the course of treatment.

Approximately one month prior to referral to our service, the patient reported progressive left-sided hearing loss, ptosis, and visual blurring. However, most notable was a severe headache that hampered her activities of daily living. Oral pain medication no longer afforded any relief. She was started on oral dexamethasone 4 mg every eight hours and referred for surgical evaluation.

Preoperative physical examination revealed multiple cranial nerve deficits: vision limited to hand movements in the right eye; complete ophthalmoplegia ('frozen globe') and ptosis on the left; left peripheral facial nerve palsy; bilateral sensorineural hearing loss; and tongue deviation to the right, suggestive of hypoglossal nerve involvement. These findings were consistent with extensive skull base involvement and multiple cranial neuropathies.

The patient has no significant prior comorbidities but developed chronic kidney disease and hypothyroidism, both attributed to prior chemotherapy and radiation exposure.

Following a multidisciplinary team-based discussion, surgical intervention was deemed the most appropriate course of action, given that the patient had already received the maximum safe doses of chemotherapy and radiation without incurring unacceptable toxicity. To further palliate her current symptoms, structural tumor debulking was recommended.

**Figure 1:**
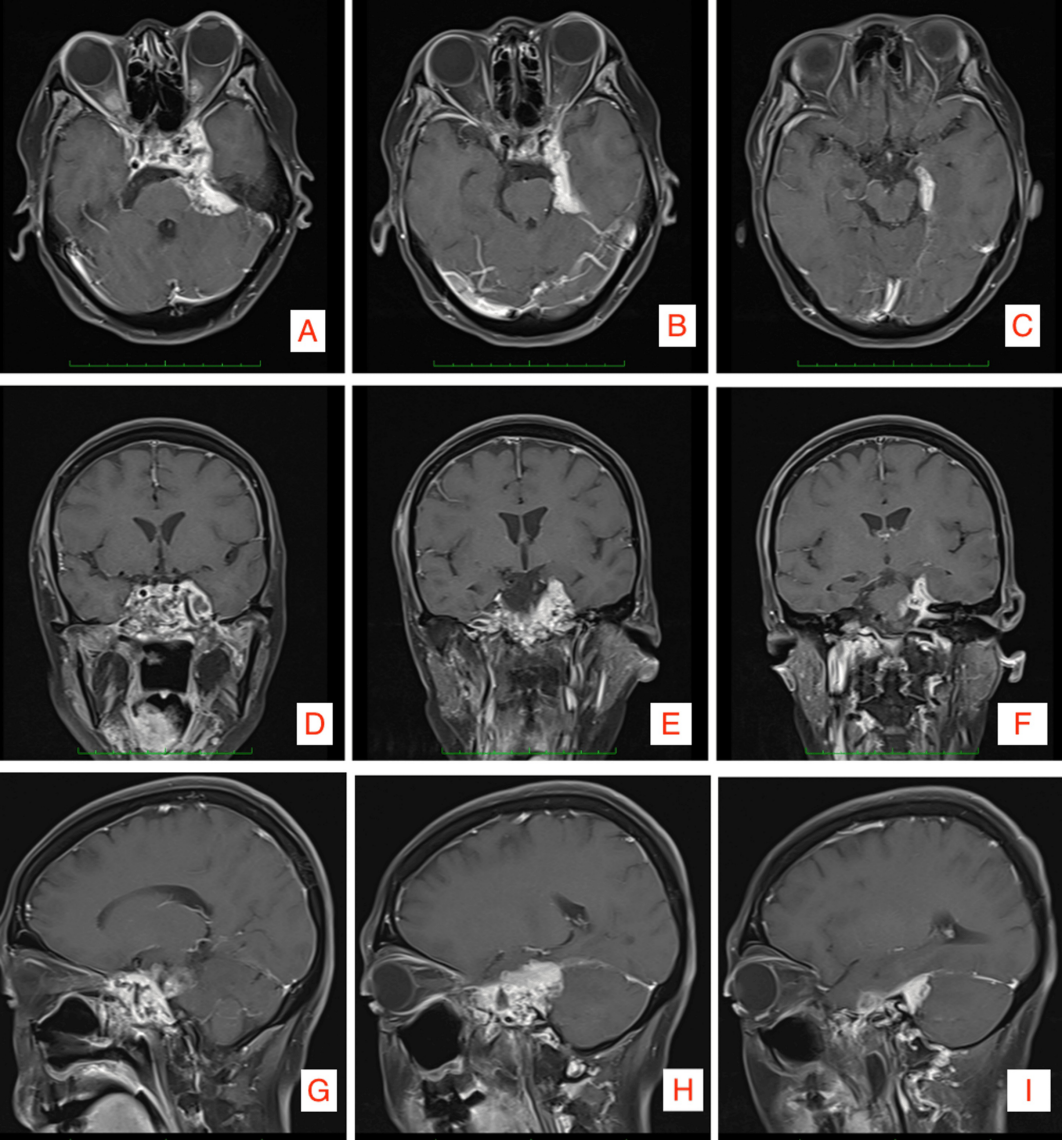
Preoperative contrast MRI. A-C: (axial view) Noted enhancing mass invading the bilateral cavernous sinus, the left antero-lateral pons, and left medial temporal lobe. D-F: (coronal view) Tumor invasion enveloping the left and right (not shown) cavernous segment of the internal carotid artery, left infero-lateral pons and uncus. G-I: (sagittal view) nasopharyngeal tumor extension to the clivus and pre-pontine cistern.

**Figure 2:**
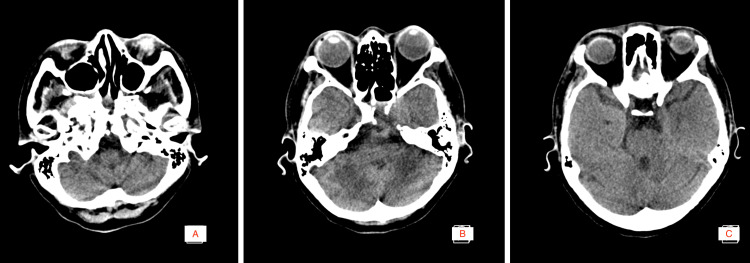
Pre-operative cranial CT scan A-C: (axial view) Bony anatomy relevant to preoperative planning denoting pneumatization of the petrous bone.

Neuroimaging was requested. Figure 1revealed that the mass invades the bilateral cavernous sinuses, extends between critical neurovascular structures, and abuts the basal cisterns. The cranial CT Scan in Figure 2highlights the bony structures. Relevant anatomical concerns include the degree of pneumatization of the petrous bone, lysis of the cavernous sinus walls and reactionary sinusitis. 

The patient underwent a left anterior and posterior petrosectomy, mastoidectomy, and retrosigmoid craniotomy for subtotal tumor excision. The primary objective of the procedure was palliative, aiming to reduce tumor burden for symptomatic relief, facilitate restaging, and discontinue chronic corticosteroid therapy. Approximately 50% of the tumor was resected. Intraoperatively, cranial nerves V, VII, and VIII, including their extensions into the posterior fossa, were identified and preserved. Residual tumor remained along the margins of the cavernous sinus, as the principal goal was to alleviate mass effect. Conservative resection and safeguarding the lateral wall of the cavernous sinus decreases morbidity by avoiding inadvertent blood loss. Given the extent and chronicity of the pathology, reversal of the cranial neuropathies postoperatively is considered highly unlikely.

The patient was successfully extubated two days postoperatively and transferred from the intensive care unit (ICU) on the fourth postoperative day. Although she demonstrated persistent left-sided extraocular muscle paralysis and peripheral facial palsy, significant improvement in facial symmetry was noted upon follow-up at four weeks.

**Figure 3:**
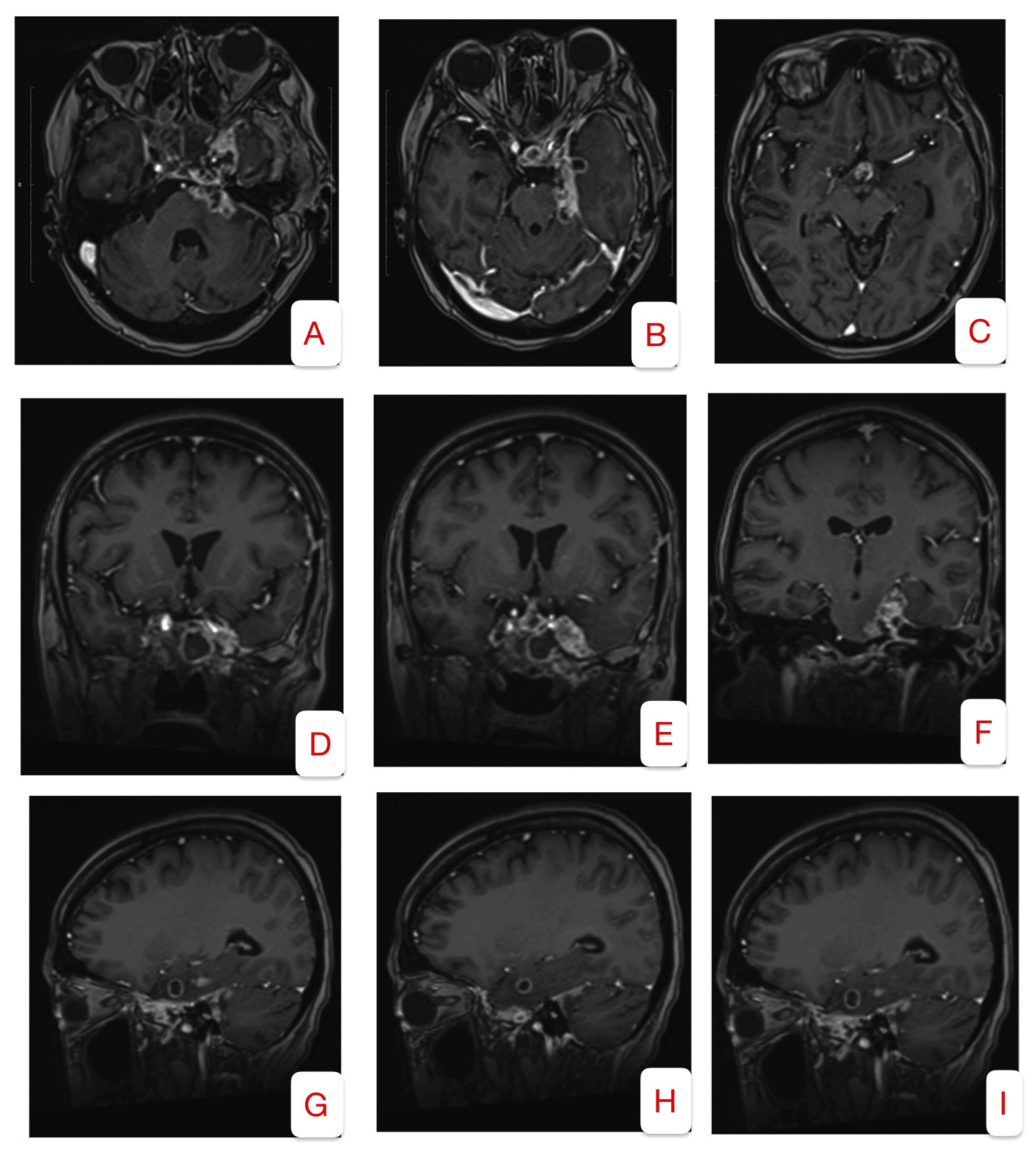
Postoperative contrast MRI A–C (Axial Views): There is a marked reduction in the size of the enhancing mass lesion involving the bilateral cavernous sinuses, the left anterolateral pons, and the left medial temporal lobe. D–F (Coronal Views): Imaging demonstrates a reduction in mass effect on the uncus, with associated improvement in vasogenic edema, as evidenced by decreased cortical effacement. G–I (Sagittal Views): The nasopharyngeal tumor continues to extend to the clivus and prepontine cistern. However, there is a notable reduction in enhancement along the tentorium, and the superior extent of the tumor has significantly decreased.

A repeat contrast-enhanced cranial MRI obtained three months postoperatively (Figure 3)demonstrated a notable reduction in tumor volume. The lesion exhibited decreased contrast enhancement, suggesting a reduction in vascularity or tumor activity. Additionally, there was improved decompression of adjacent brain structures, with diminished mass effect and resolution of surrounding vasogenic edema, indicating a favorable response to the surgical debulking and reduction of intracranial pressure.

At the six-month follow-up, the patient reported complete resolution of her previously severe headaches and a marked improvement in overall quality of life. The therapeutic goals were considered achieved, as she was able to resume daily activities and engage in remote work without the debilitating pain that had previously impaired her functional capacity. Notably, this improvement was sustained without reliance on oral analgesics or corticosteroids. As anticipated, there was minimal improvement in the existing cranial neuropathies. 

## Discussion

Management of nasopharyngeal carcinoma encompasses a broad spectrum of surgical and nonsurgical modalities. Prior to any intervention, this patient’s case was reviewed in a multidisciplinary team conference where the risks and benefits of potential treatments were thoroughly evaluated. As emphasized by Mauer et al. [3], an optimal multidisciplinary team for such complex cases should include specialists in neurosurgery, neurology, radiation oncology, medical oncology, and palliative care.

Given the patient's prior exposure to maximal doses of chemotherapy and radiation, both of which failed to yield a therapeutic response, she was referred to our service for palliative surgical management. The goal was twofold: to alleviate symptoms stemming from cranial nerve compression and to obtain tissue samples for pathological reassessment and disease re-staging.

An additional surgical objective was to reduce the risk of carotid blowout syndrome, a potentially fatal complication often observed in patients with a history of radiotherapy and head and neck surgery [4]. The carotid arterial walls and siphon are at risk due significant tumor invasion. As described by Giammattei et al. [5], this syndrome typically results from radiation-induced compromise of arterial wall integrity, rendering vessels vulnerable to rupture under normal hemodynamic pressure.

In this case, a combined transpetrosal approach was selected to provide a broad surgical corridor suitable for addressing the extensive skull base pathology. This technique, particularly when supplemented by access via the retrosigmoid route, facilitated exposure of the anterolateral brainstem and the petroclival region, enabling safe and effective resection of the lesion [6].

Several critical considerations in the surgical approach were adapted from the operative experience described by Jiang [7].

Cosmetic mastoidectomy

Beyond its aesthetic benefit of minimizing visible postoperative defects, Fava [8] emphasized that the preservation of the outer cortex during a cosmetic mastoidectomy provides additional tamponade for the fat graft against the dura, thereby reducing the risk of postoperative cerebrospinal fluid (CSF) leakage. This is a devastating and life-threatening post-operative complication. A high-speed pneumatic drill is utilized to precisely dissect and preserve the outer cortex of the mastoid bone, en bloc from the diploe and inner table. The inner table was later drilled out until dura mater was exposed.

Middle fossa dissection

**Figure 4:**
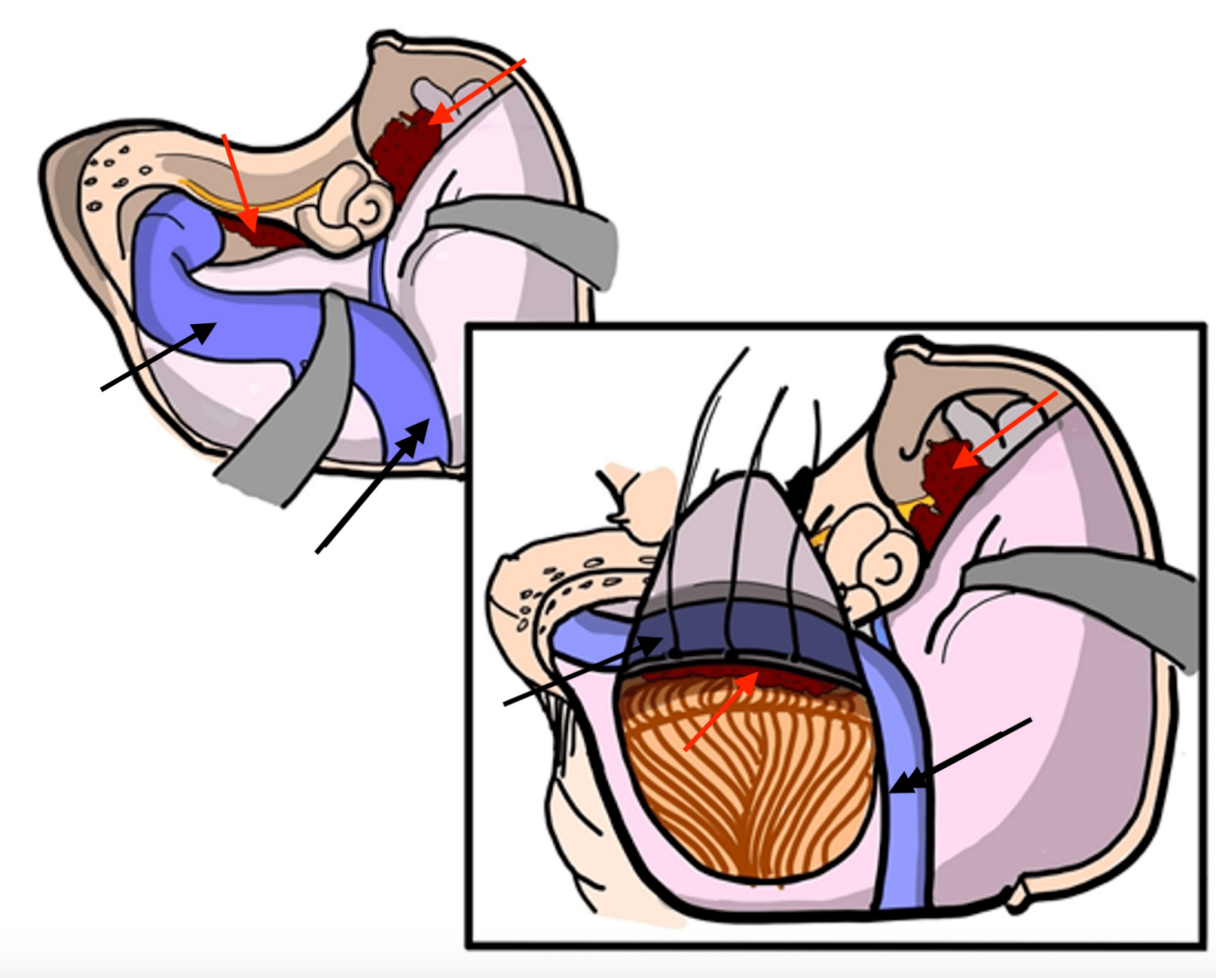
Intradural exposure of the tumor and neurovascular structures via the retrosigmoid corridor Red Arrows: Indicate intradural and extradural tumor; Single Black Arrow: Sigmoid Sinus; Double Black Arrow: Transverse Sinus Image Credits: Ian Kristopher Bayan, MD. Posted with permission.

A temporal craniotomy flushed to the floor of the middle fossa was performed. In accordance with the approach advocated by Hanakita et al. [9], extradural tumor debulking was conducted to minimize manipulation of adjacent neural structures. Following reflection of the middle fossa dura, key intradural anatomical landmarks were identified as described by Tan [10], including the middle meningeal artery, greater superficial petrosal nerve (GSPN), trigeminal nerve, arcuate eminence, and carotid canal. Early identification of these landmarks are integral to preserving these structures. An anterior petrosectomy was then performed to access the petrous apex and facilitate exposure of the cavernous sinus and Meckel’s Cave. The area of this exposure is illustrated in ​​​Figure 4* *and highlights the advantage of minimized retraction with meticulous bonework. Once sufficient decompression was achieved, the dissection proceeded to the infratentorial compartment. 

Posterior fossa dissection

**Figure 5:**
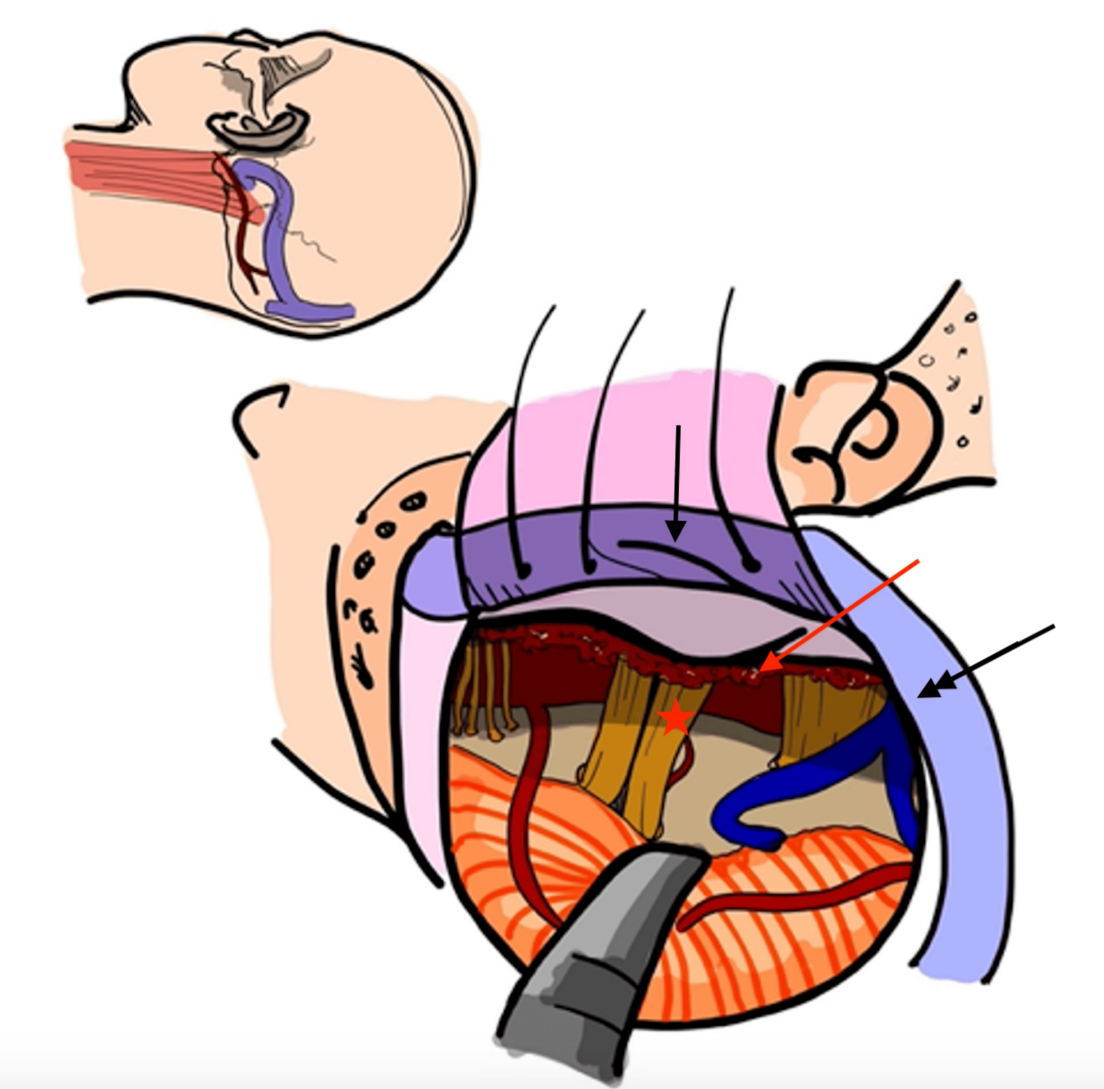
Initial extradural exposure of the petrous bone through a combined transpetrosal approach followed by intradural exposure to access the inferolateral extension of the tumor Single Black Arrow: Sigmoid Sinus; Double Black Arrow: Transverse Sinus; Red Arrow: Tumor; Red Star: CN 7/8 Complex; CN 5 is located immediately to the right of the red star Image Credits: Ian Kristopher Bayan, MD. Posted with permission.

Figure 5* *provides a representation of the surgical corridor afforded by skeletonization of the sigmoid sinus. This technique was performed as outlined by Troude et al. [11], in which after skeletonization of the sinus, this affords enough room for a limited retrosigmoid craniectomy to access the lateral infratentorial extension of the tumor. By staying close to the sinus, dura is reflected maximally, larger corridor increases the working angle between the ridge and cerebellum. Furthermore, it permits more light in the corridor for better microscopic exposure. Drilling should always be performed under high magnification and through the use of a diamond burr. Tumor surrounding the facial nerve was carefully removed to achieve facial nerve decompression to alleviate pain.

Reconstruction

Reconstructive efforts as described by Hao et al. [12] indicated that the placement of large fat grafts along the mastoid cavity and use of vascularized pedicled flaps to obliterate the surgical defect. A large part is owed to the hydrophobic properties of fat. This technique was employed to reduce the risk of pseudomeningocele formation and CSF leakage postoperatively.

## Conclusions

With expertise in skull base navigation, salvage surgery can be a viable strategy to extend survival and enhance the quality of life in patients who have exhausted conventional therapeutic options but continue to suffer from compressive symptoms. It was demonstrated that reducing tumor burden through salvage surgery can also increase the tumor's susceptibility to adjuvant systemic therapies. Furthermore, a maximal safe resection may reduce the necessity for high radiation doses, thereby aiding in the preservation of critical structures at risk.

In this instance, the combined transpetrosal approach with retrosigmoid craniectomy worked synergistically to establish a broader surgical corridor, thereby facilitating access to the extensive skull base tumor. As a result, this salvage procedure emerged as an efficacious palliative intervention, substantially improving the patient's quality of life.
